# On Obstructions of the Pulmonary Arteries

**Published:** 1844-06-29

**Authors:** James Paget


### ON OBSTRUCTIONS OF THE PULMONARY ARTERIES.

BY JAMES PAGET.

The obstructions treated of are those produced by clots of blood formed during life. They occur in nearly all cases in which the capillary circulation through a part of a lung is prevented for a considerable time before death: and this, in consequence of the arrangement of the pulmonary arteries, which do not anastomose, except at their smallest branches and in the capillary system; so that whenever any part of that system is obstructed there must be a stagnation of the blood in all the branches of the arteries leading to that part. Branches of the pulmonary arteries are usually found filled by old coagula, in cases — 1st, of compact pulmonary apoplexy; 2d, of extreme œdema of the lungs, especially in that form which is attended by peculiar rottenness of their texture, and which is apt to supervene in old persons upon disease of the heart, or emphysema after repeated attacks of bronchitis; 3d, of pneumonia with solid deposit; 4th, of cancer of the lung, when cancerous matter has been conveyed by the circulation into the branches of the pulmonary arteries. Cases of the occurrence of such coagula in these several diseases are related.

But besides these cases, in which the formation of the coagula is in a greater or less degree the consequence of obstruction of the capillaries of the lung, there are others in which it appears as the chief and primary disease.

Three such cases are detailed. In all of them numerous branches of the pulmonary arteries of the second, third, and more distant orders, were completely blocked up by coagula, which had evidently been formed long before death, and besides which no sufficent cause of death could be found. In one of the cases some of the coagula had become organized, and formed pale, firm bands and loops attached to the walls of the artery. In two of these cases there was no indication whatever of inflammation of the pulmonary artery, or its branches, having existed: they were in all respects healthy, except in having spots of yellow deposit in their coats, a change which the author states to be very common in the secondary and smaller branches of the pulmonary artery. In the last case related, there were abundant fibrinous deposits in the pulmonary valves, with wart-like growths, and ulceration of the adjacent part of the artery. There were only two valves in this pulmonary artery, and the author takes this occasion to mention the fact, that in the majority of cases in which only two valves have been found in the pulmonary artery or aorta, those valves have been diseased. He points it out as an example of a congenital defect in the shape of a part being accompanied by a more important congenital imperfection of its tissue; and alludes to the necessity of considering the latter imperfections as predisposing causes of disease in the imperfect part.

Mr. Hewitt mentioned several cases in which he had lately met with but two semilunar valves at the roots of the great arteries. In two cases there was disease—vegetation—of the valves; in a third the valves were healthy, and performed their office perfectly.

Mr. Paget observed, that in cases of valvular malformation there was greater liability to disease than usual: but he held that this was more owing to imperfection of tissue or texture, than to alteration of shape.—*Medical Gazette.*

